# Mismatch in Chronology of Environmental Cues That Initiate Spawning Increases Predation Risk for Dispersing Lake Sturgeon Larvae

**DOI:** 10.1002/ece3.72859

**Published:** 2026-01-11

**Authors:** Joseph J. Riedy, Lydia Wassink, Edward A. Baker, Kim T. Scribner

**Affiliations:** ^1^ Department of Integrative Biology Michigan State University East Lansing Michigan USA; ^2^ Michigan Department of Natural Resources Marquette Michigan USA; ^3^ Department of Fisheries and Wildlife Michigan State University East Lansing Michigan USA

**Keywords:** evolutionary trap, lake sturgeon, lunar illumination, predation risk, predator–prey dynamics, recruitment, temperature‐dependent size

## Abstract

Many species initiate reproductive activities, including migration and reproduction, in response to environmental cues that have historically been predictive of physical and biotic conditions favorable for offspring survival. Climate change can cause environmental variables including temperature levels and variability to become unreliable reproductive cues, potentially creating an evolutionary trap. For stream fishes inhabiting temperate environments, spawning may start at temperatures historically associated with high offspring survival; however, realized survival can be diminished due to mismatches with other ecological factors. We experimentally examined how an induced mismatch among historically temporally autocorrelated or matched stream physical and biotic environmental conditions present during lake sturgeon ( *Acipenser fulvescens* ) spawning and larval development and dispersal influenced larval predation rates. We used an artificial stream mesocosm to quantify the influences that matched or mismatched combinations of environmental variables including larval size (determined by incubation temperature), density of co‐occurring prey species, lunar illumination levels, and interactions among factors had on larval lake sturgeon survival when exposed to rock bass ( *Ambloplites rupestris* ), a common stream predator. We documented comparatively higher survival during matched conditions when drifting prey density was high and larval body size was large, and when dispersal occurred during periods of simulated low lunar illumination. Survival rates decreased when the combination of ecological factors lake sturgeon larvae experienced was mismatched, and therefore, supported the prediction that lake sturgeon are vulnerable to a climatically induced evolutionary trap. Results suggested changing proportional contributions to population recruitment from early and late spawning lake sturgeon may be expected during years of matched compared to environmentally mismatched spawning conditions. Data supporting management efforts for species of conservation should be examined to determine the extent to which mismatched ecological conditions influence larval survival.

## Graphical abstract

Many species rely on temporally autocorrelated environmental cues that are predictive of conditions favorable to offspring survival to initiate reproduction. Climate change can cause cues to become unreliable, creating an evolutionary trap. Mesocosm raceway experiments were used to quantify the influences of stream physical and biotic factors and behavior, specifically night light level, rearing temperature (affecting larval body size), and abundance of alternative prey, on levels of larval lake sturgeon predation during simulated downstream migration.

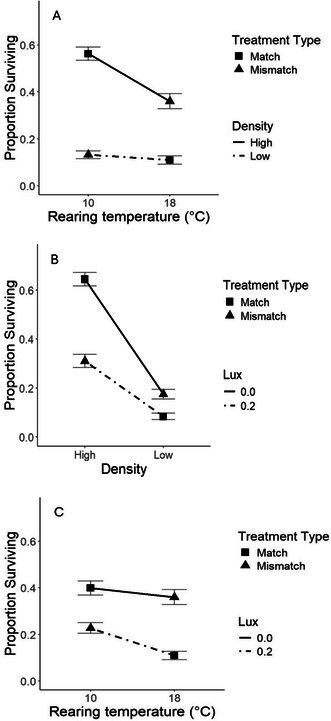

## 1 Introduction

Organisms inhabiting temperate aquatic environments use multiple environmental cues, including changes in temperature, precipitation, photoperiod, and nightly light conditions coincident with the lunar phase to guide the timing of life events (Iwasa and Levin 1995; Kumar et al. 2022). Physiological responses to environmental cues coordinate migrations to suitable habitat for mating (Forsythe, Crossman, et al. 2012; Larson et al. 2025; Post et al. 2001), and synchronize gonadal and gamete maturation, and intra‐ and inter‐sexual mating behaviors (Kumar et al. 2022; Takemura et al. 2004). For fitness outcomes of responses to environmental cues to be adaptive, cues should predict, or match, favorable ecological conditions during offspring early life stages. Such conditions have been shown to increase survival due to decreased predation and increased availability of food resources (Durant et al. 2007; Laurel et al. 2021; Levitan et al. 2011).

Climate change has caused historically predictable environmental cues to become increasingly variable in terms of timing and magnitude (Robertson et al. 2013). Global warming is causing many aquatic species to reproduce earlier in the year due to rising water temperatures earlier in the reproductive period (Koenigbauer et al. 2025; Lyons et al. 2015; Tao et al. 2018; Table S1). While some species may be able to adapt, earlier spawning can have detrimental population effects by creating a phenological mismatch with offspring food sources and increasing vulnerability to predators and environmental stressors (Bonzi et al. 2024; Donelson et al. 2014; Hovel et al. 2017). Earlier periods are also frequently characterized by increased temperature variability, even on a diel temporal scale. If current climatic warming trends continue, mismatches between temperature and resource availability and requirements during early life stages are predicted to arise for many species (Laurel et al. 2021). This is an example of an evolutionary trap (Schlaepfer et al. 2002), where a once adaptive response (e.g., onset of reproduction) to a formerly reliable environmental cue (e.g., temperature) can become detrimental due to declines in offspring survival. It is particularly important to understand whether species of conservation concern that rely on environmental cues to initiate reproductive activities may be susceptible to an evolutionary trap due to climate change that could compound with unanticipated consequences (Peer and Miller 2014).

Lake sturgeon ( *Acipenser fulvescens* ) is a long‐lived primitive teleost fish species of conservation concern throughout much of its range (Bruch et al. 2016). Lake sturgeon rely on environmental cues including temperature, river discharge, and lunar phase to time spawning migrations up rivers and to initiate spawning (Forsythe, Crossman, et al. 2012). Within each year, there are distinct lake sturgeon spawning periods, each occurring at different temperature ranges as spawning rivers warm in the spring (Duong et al. 2011; example of spawning chronology and stream temperature for lake sturgeon shown in Figure S1 based on Larson et al. (2025)). Adults spawn during one spawning period within a year (Larson et al. 2020), and the location and timing of spawning for individual adults is repeatable across years (Forsythe, Scribner, et al. 2012).

Photoperiod has also been widely documented to be an important environmental cue associated with spawning initiation in fish species (e.g., Bromage et al. 1984). Photoperiod is widely documented to affect fish gamete growth (Hansen et al. 1992), maturation (Imsland et al. 1997), and timing of reproduction (Akhoundian et al. 2020). However, specific effects of photoperiod depend on the species and its breeding cycle under natural or aquacultural conditions. For example, in the case of lake sturgeon, long‐term research (summaries in Duong et al. 2023; Forsythe, Crossman, et al. 2012; Larson et al. 2025) has shown that while photoperiod and stream temperatures are correlated, increasing water temperature, lunar phase (new moon), and declining stream discharge are the primary influences to initiation of spawning and spawning duration but not photoperiod. Data collected for over two decades for one well studied lake sturgeon population have documented earlier mean spawning initiation dates and greater inter‐annual variability in spawning initiation dates as well as increasing spawning period length and inter‐annual variability (summarized in Table S1 from Duong et al. (2023) for 2001–2007 and Larson et al. (2025) for 2016–2022).

Lake sturgeon larvae produced during early versus late portions of the spawning period develop at different temperatures that result in different developmental rates and size at hatch and at the onset of exogenous feeding (Atkinson 1994). When exogenous feeding begins, larvae emerge from river substrate at night and passively disperse downstream alongside other co‐distributed larval vertebrate and macro‐invertebrate members of the stream community to depositional nursery rearing habitat downstream (Auer and Baker 2002; Receveur et al. 2022; Table 1). Larval lake sturgeon produced during either earlier or later portions of the spawning period develop and migrate downstream under different environmental conditions (Duong et al. 2011) and at different body sizes (Crossman 2008). Larval lake sturgeon produced by spawning early in colder water are larger in size (Atkinson 1994; Crossman 2008; Table 1), in part because more energy from yolk sac reserves can contribute to somatic growth instead of respiration (Jonsson et al. 2022). Larval lake sturgeon produced early in the spawning period drift downstream with a more abundant and taxonomically diverse co‐distributed community of larval fish and insects than larval lake sturgeon drifting later in the dispersal period (Table 1). Larvae drifting during both dispersal periods are predated upon by a lake sturgeon predator assemblage that does not substantially change during this relatively short dispersal period (Receveur et al. 2022; Waraniak et al. 2019; Table 1). Additionally, larvae produced during the early spawning period are more likely to drift during darker conditions of a new moon because early spawners use the new moon as a cue to begin spawning (Forsythe, Crossman, et al. 2012; Table 1). Lake sturgeon produced during the early spawning period take approximately 30 days post fertilization, or one lunar phase cycle, to reach the exogenous feeding stage and begin downstream dispersal (Duong et al. 2011). During downstream drift, larvae are exposed to taxonomically diverse fish predators and mortality is high (Waraniak et al. 2018), though mortality levels have not been empirically evaluated under matched versus mismatched ecological conditions.

**TABLE 1:** Ecological conditions at the time of lake sturgeon spawning are associated with “matched” conditions experienced by larvae during passive downstream dispersal from spawning to rearing areas that affect larval predation risk.

Environmental conditions	Matched conditions associated with larval dispersal following early spawning period	Matched conditions associated with larval dispersal following late spawning period	References
Lunar illumination (phase)	Dispersal during periods of lower lunar illumination (new moon; under the cover of darkness)	Light levels more variable during dispersal (during nights with higher lunar light levels)	Waraniak et al. (2019); Forsythe, Crossman, et al. (2012)
Stream benthic dispersing community composition and abundance, and predation risk	Larvae disperse (shielded among) diverse abundant co‐distributed larval fish and macroinvertebrate taxa	Larvae disperse with fewer and less diverse co‐distributed larval fish and macroinvertebrate taxa	Receveur et al. (2022)
Larval body size at hatch associated with water temperature during early and late spawning periods (Figure S1)	Large larval lake sturgeon associated with colder incubation conditions	Small larval lake sturgeon associated with warm temperature conditions	Atkinson (1994); Crossman (2008)

Our objective was to quantify and compare larval lake sturgeon survival rates in mesocosm predation experiments under combinations simulating ecological factors set to match or mismatch the ecological stream conditions present during the early and late spring periods of spawning and larval dispersal. Experiments demonstrate the individual adult fitness consequences of maladaptive spawning timing in response to increasingly unreliable combinations of environmental cues that are mismatched to larval environmental conditions, forecasting future susceptibility to climate‐induced evolutionary traps (sensu Schlaepfer et al. 2002).

## 2 Materials and Methods

### 2.1 Lake Sturgeon Rearing

Spawning lake sturgeon were captured in the river, and their gametes were collected streamside to produce the larvae used in our experiments. Eggs from a single female were fertilized with sperm from a single male to produce full‐sibling families. Each female and male were used to produce only one family. All fertilizations, lake sturgeon rearing, and subsequent experiments took place in a streamside rearing facility adjacent to the river which supplied river water at ambient temperatures. Four full‐sibling families were produced in 2018 and four additional full‐sibling families were produced in 2019. Fertilized eggs were disinfected with povidone‐iodine (Betadine, Stamford, CT), and were treated with Fuller's earth for de‐adhesion, following established lake sturgeon hatchery protocols (Bauman et al. 2016; Crossman et al. 2011). After fertilization, eggs from each female were evenly divided into two incubation temperature exposure groups, resulting in eight replicated families within each temperature exposure group. Fertilized eggs from one group were incubated in Heath trays at 10°C, simulating temperatures present early in the spawning season, while eggs from the other group were reared at 18°C, representative of temperatures late in the spawning season (see Figure S1 for illustration of spawning chronology and environmental features during one spawning season). Incubation temperatures were expected to result in relatively larger and smaller body size, respectively at hatch (Atkinson 1994; Crossman 2008; Table 1). Following hatch, free embryos from each group were reared in a temperature controlled partially recirculating system supplied with 50 μm‐filtered water pumped from the river. During egg incubation and post‐hatch free embryo periods, rearing containers were equipped with a heater and chiller to allow for temperature control to maintain 10°C and 18°C temperatures (1800 W Single Tube Bottom Heater, Pentair AES, Apopka, FL; Arctica ¼ HP 1000 W Aquarium Chiller, Transworld Aquatic Enterprises, Inglewood, CA). At hatch, yolk sac larvae were transferred to 3 L tanks (Aquatic Habitats; Pentair, Apopka, FL) containing 32 bio‐balls (CBB1‐S; Pentair, Apopka, FL) that simulated spawning stream substrate. Tanks held up to 100 lake sturgeon yolk‐sac larvae. Within 24 h of emergence from artificial substrate and transitioning into the exogenous feeding larval stage, individuals were placed in 3 L tanks containing no bio‐balls, and were used in experimental predation trials. These tanks contained either 10°C or 18°C water depending on the treatment groups, but were supplied with water lines introducing ambient‐temperature river water to allow the lake sturgeon larvae to acclimate to the water temperature of the predation experiments. Ten individuals of each family and incubation temperature combination were photographed using a digital camera with a ruler, and body length was measured using ImageJ software (National Institute of Health, Bethesda, MD).

### 2.2 Predation Experiment

Predation experiments were designed and predatory rock bass ( *Ambloplites rupestris* ) were handled following protocols established by Waraniak et al. (2017) as updated by Riedy et al. (2025). Water flow through the mesocosm raceways was used to simulate conditions experienced by drifting lake sturgeon larvae in the river (Receveur et al. 2022). Rock bass, a common stream predator known to prey on larval lake sturgeon (Waraniak et al. 2019) were captured via electrofishing from the stream and transported to the streamside rearing facility in aerated coolers filled with ambient temperature river water. Rock bass were held in 3.58 × 0.5 m tanks supplied with water pumped from the river at the ambient river temperature for 24–36 h prior to predation trials as described previously (Riedy et al. 2025). Two hours before a trial began, two rock bass were placed sequentially in separate gated sections (one upstream of the other) of a 7.15 × 0.5 m raceway, to allow predators to acclimate to the mesocosms while preventing aggressive interactions between individuals. Raceways were supplied with ambient temperature river water (range over the experimental period 16.8°C–25.2°C) over both years of the study. One hour before trials, the water velocity was increased to 0.134 ± 0.003 m/s in the raceways to simulate stream flow rates larval lake sturgeon experience during the larval dispersal period (Receveur et al. 2022). At this time, the light level in the building housing the raceways was set to either 0.00 lux, simulating new moon illumination conditions that are typically present during the dispersal period of lake sturgeon larvae produced early in the spawning season, or 0.20 lux, simulating a moon lit night that is more likely to be experienced by larvae produced later in the spawning season (Forsythe, Crossman, et al. 2012; Table 1). Water velocity was measured using a Marsh‐McBirney Flo‐Mate 2000 flow meter (Frederick, MD), and lux was measured using an Extech LT300 light meter (Waltham, MA).

At the beginning of each trial, small mesh nets were placed over raceway outflows, and co‐dispersing larvae were released into the upstream end of the raceways. Equal numbers of larval lake sturgeon, larval white suckers ( *Catostomus commersonii* ), and Heptageniid mayflies were released simultaneously at the beginning of each trial to simulate the co‐distributed prey community present during the larval drift period (Receveur et al. 2022; Waraniak et al. 2019). The lake sturgeon used in a single trial were from the same full‐sibling family and temperature incubation group. White sucker larvae and Heptageniid mayfly larvae were captured dispersing in the natal river using D‐frame nets deployed at night, and via kick‐netting of stream substrate. Additional individuals of the three prey taxa were released 15, 30, and 45 min after the beginning of the trial. Each pulse of prey released contained either eight individuals of each taxon in trials simulating high prey density early in the lake sturgeon larval drift period, or two individuals of each taxon for trials simulating comparatively lower prey densities in the later period. This design resulted in a total of either 32 or 8 individuals of each taxon (high and low density treatments, respectively; Receveur et al. 2022; Table 1) released per trial. After 1 h, predators were removed from the mesocosms, water flow was reduced, and the number of surviving lake sturgeon larvae swimming in the raceway and captured in out‐flow nets was captured using short‐handled dip nets and counted. Within each family, a trial was conducted for every unique rearing temperature group by light level and drifting prey density combination, resulting in 8 trials per family and 64 trials total.

### 2.3 Statistical Analysis

We used a binomial family generalized linear mixed effect model to quantify the proportion of lake sturgeon surviving predation trials as a function of fixed effects of rearing temperature (and associated larval lake sturgeon body size), light level, prey density, as well as the two‐way interactions between these effects. To determine whether the ambient water temperature during the raceway predation trails influenced the sturgeon survival rate, we conducted an additional mixed effect generalized linear model including the same effects as the previous model, with the addition of ambient water temperature. Additionally, we used a linear mixed effect model to determine the effect of rearing temperature on lake sturgeon total length at the beginning of the larval stage. Both models included the random effect of family. Models were produced in R v 4.2.0 using packages lme4 and MuMIn. Diagnostic checks of the models were conducted using the DHARMa package to ensure simulated residuals were uniform, not over‐dispersed, and were not zero‐inflated.

## 3 Results

All the fixed effects and interactions included in binomial family generalized linear mixed effect model were statistically significant (Table 2), and larval body size was significantly related to rearing temperature (*p* < 0.001). Larvae reared at 18°C were smaller on average than those reared at 10°C (19.1 vs. 21.9 mm total length; *p* < 0.001). When the model was run again adding ambient raceway water temperature during the predation trials as an additional fixed affect, raceway water temperature was not significant (*p* = 0.41). Additionally, model diagnostics using the DHARMa package indicated there was no significant deviation from expected patterns (test for uniformity: *p* = 0.682; overdispersion: *p* = 0.384; zero‐inflation: *p* = 0.999), and therefore model assumptions had been met.

**TABLE 2:** Parameter estimates (log odds), standard error, and *p*‐values for the terms included in the binomial generalized linear mixed effect model describing the proportion of lake sturgeon larvae surviving predation trials.

Parameter	Estimate	Std. error	*p*
Intercept	0.7991	0.1431	< 0.001
Rearing temp. 18	−0.4751	0.1696	0.005
Density low	−2.4121	0.1776	< 0.001
Lux 0.2	−1.0928	0.1563	< 0.001
Rearing temp. 18: Density low	0.6166	0.2318	0.008
Rearing temp. 18: Lux 0.2	−0.7027	0.2247	0.002
Density low: Lux 0.2	0.5566	0.2316	0.016

Larval lake sturgeon reared at 18°C had significantly lower survival rates during predation trials than those reared at 10°C (Table 2; *p* = 0.005). Larval lake sturgeon drifting with low densities of co‐distributed prey taxa also had significantly lower survival rates (Table 2; *p* < 0.001). Also, larval lake sturgeon in trials with the light level imitating a full moon had lower survival rates than in trials with lower levels of light (Table 2; *p* < 0.001). The variance in larval lake sturgeon survival attributed to the random effect among families was estimated to be 0.053 (1.6% of the remaining variance) by the mixed effect model.

**FIGURE 1:**
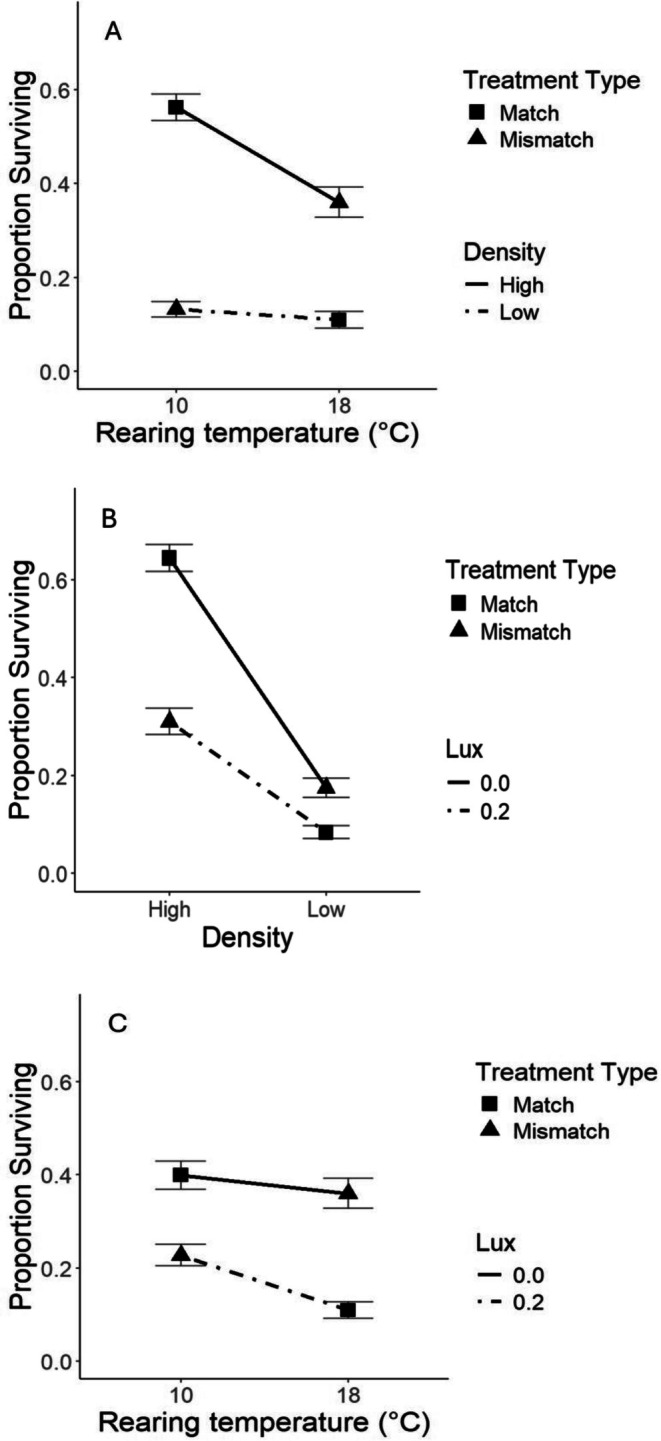
The proportion of larval lake sturgeon surviving in experimental mesocosm predation trials when exposed to predatory rock bass. Points and error bars reflect predictions and 95% confidence intervals produced by the model indicated in Table 2 under matched and mismatched environmental conditions (Table 1). (A) The interaction between the effects of prey taxa density included in trials and lake sturgeon rearing temperature. (B) The interaction between light level (lux) during trials and prey taxa density. (C) The interaction between light level and lake sturgeon rearing temperatures.

The interaction between density of co‐distributed prey and incubation temperature (a determinant of larval body size) was significant (Table 2; *p* = 0.008). Rearing temperature did not have a strong influence on survival rates of lake sturgeon larvae drifting with low densities of co‐distributed prey, however at high densities, larger larvae reared at 10°C had higher survival than those reared at 18°C (Figure 1A). The interaction between light level and co‐distributed prey density was also significant (Table 2; *p* = 0.002). The effect of density was greater at 0.0 lux than at 0.2 lux (Figure 1B). Specifically, larval lake sturgeon dispersing with a low density of co‐distributed prey experienced lower survival rates during new moon conditions than during full moon conditions. Lastly, the interaction between light level and rearing temperature was also significant (Table 2; *p* = 0.016). During new moon conditions, rearing temperature did not have a strong influence on the survival rates of larval lake sturgeon in predation trials, however in full moon conditions, larvae reared at 10°C had higher survival rates than the smaller lake sturgeon reared at 18°C (Figure 1C).

## 4 Discussion

Evolutionary traps can occur when historically adaptive responses to an environmental cue become maladaptive due to environmental changes, leading to mismatched environmental and ecological conditions (Table 1) experienced by individuals at the same or different ontogenetic stages (Robertson et al. 2013). Life histories of aquatic poikilothermic organisms are closely tied to the environments they inhabit. Species may therefore experience an evolutionary trap when climate change makes historical temperature and lunar cycle cues for initiation of reproduction unreliable, leading to mistimed breeding that is mismatched with environmentally optimal conditions for offspring. For lake sturgeon, decadal changes in mean and standard deviation in spawning initiation dates and mean and standard deviation in duration of the spawning period (Table S1) document adult behavioral and physiological responses to climate‐mediated changes in environmental conditions that experimental results documented here revealed are associated with decreased offspring survival.

Experimental results reported here document levels of larval lake sturgeon survival after exposure to predators associated with the effects of each ecological factor tested (prey density, nocturnal light levels, incubation temperature [a surrogate for larval body size]) and their interaction during simulated larval dispersal. Each interaction in the binomial mixed effect model was significant (Table 2), demonstrating the complex relationships among ecologically relevant physical and biotic stream conditions (Alix et al. 2020; Laurel et al. 2021), and potential consequences in natural and managed systems (Brown et al. 2024; Ferreira et al. 2023; Rogers and Dougherty 2019). It is important to understand how these factors could influence lake sturgeon in riverine environments where seasonal spawning cues become unreliable predictors of larval survival. Larval lake sturgeon are likely to encounter abnormal and asynchronous combinations of physical and biotic factors more frequently during critical early developmental periods, including the larval dispersal stage simulated in these experiments (see also Receveur et al. 2022; Waraniak et al. 2019).

### 4.1 Effects of Temperature and Larval Lake Sturgeon Body Size

Lake sturgeon eggs and larvae produced early in the spawning season would historically have experienced comparatively cool incubation and development temperatures (Crossman 2008; Table 1). Larvae produced during the early portion of the spawning season passively disperse downstream concurrently with higher densities of co‐dispersing prey than larvae produced later in the spawning season (Receveur et al. 2022; Waraniak et al. 2019; Table 1). Further, large macroinvertebrate taxa that are preferred by fish predators (Waraniak et al. 2019) including Heptageniidae, Ephemerellidae, and Isonychiidae, and Catastomid fish larvae are present in greater abundance (and biomass) during early periods of drift compared to later periods (Receveur et al. 2022; Waraniak et al. 2019). Climate change may cause spring temperatures to increase more quickly and earlier (Hoegh‐Guldberg and Bruno 2010), resulting in larvae produced earlier in the spring spawning season (e.g., Koenigbauer et al. 2025 for other fish species and early April rather than late April or early May for lake sturgeon, Figure S1, Table S1). Eggs and free embryos that develop under warmer conditions would be smaller in body size during drift (Duong et al. 2011). Lake sturgeon larvae reared at 10°C were approximately 14% larger than those reared at 18°C in our experiments and had higher survival rates (Table 2). Larger prey often experience lower rates of predation due to longer handling time by predators (Nilsson and Brönmark 2000), or by being more adept at evading predation as swimming velocity and larval size are correlated (Fisher et al. 2000).

Temperature treatments evaluated here under experimental conditions were based on long‐term data (Forsythe, Scribner, et al. 2012; Table S1) but could vary in some lake sturgeon rivers. Climate change will likely result in higher average water temperatures as well as an increase in temperature variability. However, there is potential for muted changes in river thermal regimes that could lead to different outcomes, for example under different regulated river discharge conditions or if spring discharge is derived from snow melt (Oyinlola et al. 2025). Although counterintuitive, it is also plausible that after warming quickly in the spring, spawning streams may cool shortly after the later spawning season occurs, and the offspring would develop under relatively cool conditions. Detailed, long‐term monitoring and modeling of individual river systems are needed to accurately predict local thermal regimes and their impacts on lake sturgeon (Embke et al. 2023) and other species. Further research could profitably explore relationships with other stream variables that may affect river temperature and the potential for mismatch conditions that lead to evolutionary traps including altered hydrology, anthropogenic modifications such as loss of riparian shade, and groundwater inputs.

An additional factor that could potentially lead to mismatched conditions under future climate change scenarios involves anticipated shifts in the timing of initiation of spawning and expected accompanying increased variability in early season river temperatures and flow regimes (e.g., Table S1) that have been shown to interrupt spawning (Forsythe, Crossman, et al. 2012; Dammerman et al. 2019) when the largest groups of adults are in the spawning areas. Dammerman et al. (2019) documented that female reproductive success was tied to depensatory effects of the number of mates and male‐biased sex ratio; both associated with fertility assurance. Females spawning later in the season had higher per capita reproductive success, but the numbers of early‐spawning adults are much greater (Table S1). In terms of population‐wide levels of recruitment, early spawning females are much more likely to encounter temperature and flow‐related episodes of sub‐optimal conditions that will episodically decrease the number of spawning adults (Figure S1) and are predicted to decrease female reproductive success (Dammerman et al. 2019).

### 4.2 Effects of Co‐Distributed Prey

The effect of co‐distributed prey density had the largest influence on survival rate (Table 2). Results are likely to be due to predator swamping, where prey availability exceeds the maximum consumptive rate of predators, decreasing the probability that an individual will be consumed (Furey et al. 2016). Predator swamping is a strategy to escape predation that has been documented in insects, birds, reptiles, and fish (Corkum et al. 2006; Descamps 2019; Furey et al. 2016; Tucker et al. 2008). If survival rates of dispersing lake sturgeon larvae are influenced by predator swamping, the timing of spawning is important because lake sturgeon larvae drifting concurrently with other fish and invertebrate larvae will realize higher survival.

The interaction between prey drifting density and rearing temperature (Figure 1A) indicates the potential for a decrease in larval lake sturgeon survival rates when reared at 18°C relative to those reared at 10°C when drifting with a high density of co‐distributed prey. Small body size has previously been shown to negatively impact survival rates of lake sturgeon larvae (Crossman et al. 2018). However, if the reverse were to take place, and lake sturgeon produced later in the season and drifting with low densities of co‐dispersing prey taxa were larger due to rearing at lower temperatures (Figure 1), results suggest there would be little change in survival rates because rearing temperature does not strongly influence survival rates when lake sturgeon larvae are drifting with low densities of co‐distributed taxa. Further research could importantly explore the effects of climatically induced changes in stream conditions on other co‐distributed drifting prey. Importantly, climate change has been shown to significantly alter the life cycles of aquatic macroinvertebrates primarily through increased water temperature and altered flow regimes, which drive changes in development rates, emergence timing, body size, and species composition (review in Bonacina et al. 2023).

### 4.3 Effects of Nocturnal Light Conditions

Lake sturgeon use periods of low lunar illumination (new moon phase) as one primary cue to initiate spawning at the beginning of the reproductive season (Forsythe, Crossman, et al. 2012). Lake sturgeon produced at early season temperatures take roughly 30 days post fertilization to reach the exogenous feeding stage (Duong et al. 2011). Therefore, larvae produced early in the spawning season are often drifting during the next period of low lunar illumination (Receveur et al. 2022). If water temperatures increase after the onset of spawning, the time it takes larvae to develop to the exogenous feeding stage will be decreased, resulting in lake sturgeon larvae drifting under full moon light conditions. Duong et al. (2011) found that lake sturgeon developing in the egg and yolk sac larval stage at mean daily temperature of 18.3°C reached the exogenous feeding larval stage 13.5 days earlier on average than lake sturgeon developing at 15.4°C. Thus, relatively minor increases in temperature can have a large impact on the timing of lake sturgeon larval drift downstream. The interaction between light level and co‐distributed prey density (Figure 1B) indicates that if lake sturgeon larvae dispersed early in the season, at high densities, and disperse under full moon conditions instead of new moon conditions, we would expect decreased larval survival rates (Figure 1B). This result provides evidence that lake sturgeon could be susceptible to an evolutionary trap, specifically elevated levels of larval mortality due to predation, induced by increasingly mismatched physical and biotic factors between the time of spawning and egg incubation and larval development.

Light level influenced larval lake sturgeon survival, likely due to predatory rock bass, a visual predator, having difficulty in detecting prey in darker conditions. Night light level also has been associated with fish predation rates in other riverine species (Furey et al. 2016). Waraniak et al. (2018) found lake sturgeon DNA in the diet of visual predators including centrarchids, shiners, and percids, but did not find sturgeon DNA in the diet of predators that use other senses to locate and attack prey such as bullhead (*Ameiurus* spp.) and suckers (Catastomidae). Although predation levels of non‐visual larval lake sturgeon predators were not experimentally evaluated in this study, riverine fish communities do include an abundance of visual predators, and thus the effect of night light level likely has an important influence on predation levels of drifting larval lake sturgeon.

## 5 Conclusions

In conclusion, larval lake sturgeon survival rates during mesocosm experiments simulating the larval drift period were affected by light level, the density of co‐distributed alternative prey taxa present during the experiment, and egg and larval rearing temperature (a primary determinant of offspring body size). The interactions between these effects indicate larval survival rates may decrease if historically temporally autocorrelated environmental cues become mismatched, causing larval dispersal to occur during sub‐optimal physical and biotic stream conditions. As climate change influences temperatures and precipitation levels (van der Wiel and Bintanja 2021), temperature as a cue may become maladaptive and detrimental to offspring survival and levels of recruitment. Results suggest changing proportional contributions to population recruitment can be expected from early and late‐spawning lake sturgeon during years of matched compared to environmentally mismatched spawning conditions. Changes in relative reproductive contributions could have negative consequences to population persistence given the differences in numbers of early and late period spawners (Figure S1). Future research efforts for lake sturgeon and other fish and wildlife species could profitably investigate the environmental drivers, biological mechanisms, and long‐term population consequences of differential recruitment success among adults that reproduce over extended breeding periods.

## Author Contributions

**Joseph J. Riedy:** conceptualization (lead), data curation (lead), formal analysis (lead), investigation (equal), methodology (equal), visualization (lead), writing – original draft (lead), writing – review and editing (equal). **Lydia Wassink:** conceptualization (supporting), data curation (supporting), formal analysis (supporting), investigation (supporting), methodology (supporting), writing – review and editing (supporting). **Edward A. Baker:** funding acquisition (supporting), investigation (supporting), project administration (supporting), resources (supporting), writing – review and editing (supporting). **Kim T. Scribner:** conceptualization (supporting), funding acquisition (supporting), investigation (supporting), project administration (lead), resources (lead), supervision (supporting), writing – review and editing (supporting).

## Funding

This work was supported by the Michigan Department of Natural Resources, State Wildlife Grants Program.

## Ethics Statement

All procedures associated with the research reported were conducted under protocols approved by the Michigan State University Animal Use and Care Committee under permit number 05/16‐056‐00.

## Conflicts of Interest

The authors declare no conflicts of interest.

## Supporting information


**Figure S1:** Numbers of adult male and female lake sturgeon present in the river spawning areas by day during the 2019 spawning season.

**Table S1:** Time series summarizing lake sturgeon spawning initiation date and spawning duration during two time periods including the 7 year periods described by Duong et al. (2023) (figure 2; 2001–2007) and by Larson et al. (2025) (figure 2; 2016–2022).

## Data Availability

All data, metadata, R‐code for statistical code are available at the Zenodo repository at https://doi.org/10.5281/zenodo.15844213.
