# Supplementary figures and images for: Mismatch in Chronology of Environmental Cues That Initiate Spawning Increases Predation Risk for Dispersing Lake Sturgeon Larvae
Source: Ecol Evol. 2026 Jan 11;16(1):e72859. doi: 10.1002/ece3.72859 (PMC12790875; doi:10.1002/ece3.72859)

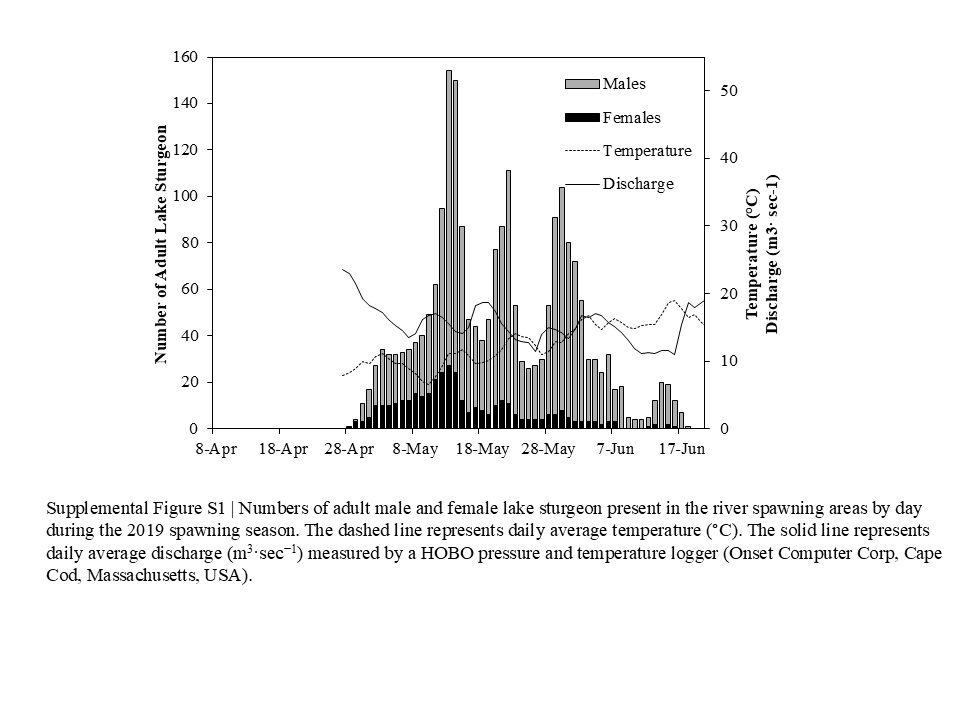

Supplement: Supplementary file 1 — Figure S1: Numbers of adult male and female lake sturgeon present in the river spawning areas by day during the 2019 spawning season. [file ECE3-16-e72859-s001.tif]
